# New Senolysis Approach via Antibody–Drug Conjugate Targeting of the Senescent Cell Marker Apolipoprotein D for Skin Rejuvenation

**DOI:** 10.3390/ijms24065857

**Published:** 2023-03-20

**Authors:** Kento Takaya, Toru Asou, Kazuo Kishi

**Affiliations:** Department of Plastic and Reconstructive Surgery, Keio University School of Medicine, Tokyo 160-8582, Japan

**Keywords:** aging, senolysis, antibody drug conjugate, apolipoprotein D

## Abstract

Senescent cells accumulate in aging skin, causing age-related changes and a decline in functional efficiency. Therefore, senolysis, a treatment that specifically removes senescent cells and rejuvenates the skin, should be explored. We targeted apolipoprotein D (ApoD), a previously identified marker expressed on senescent dermal fibroblasts, and investigated a novel senolysis approach using a monoclonal antibody against this antigen and a secondary antibody conjugated with the cytotoxic drug pyrrolobenzodiazepine. Observations using fluorescently labeled antibodies revealed that ApoD functions as a surface marker of senescent cells and that the antibody is taken up and internalized only by such cells. The concurrent administration of the antibody with the PBD-conjugated secondary antibody specifically eliminated only senescent cells without harming young cells. The antibody–drug conjugate treatment of aging mice combined with the administration of antibodies reduced the number of senescent cells in the dermis of mice and improved the senescent skin phenotype. These results provide a proof-of-principle evaluation of a novel approach to specifically eliminate senescent cells using antibody–drug conjugates against senescent cell marker proteins. This approach is a potential candidate for clinical applications to treat pathological skin aging and related diseases via the removal of senescent cells.

## 1. Introduction

The accumulation of senescent cells in tissues causes a number of age-related medical conditions [1]. In recent years, pharmacological research aimed at eliminating senescent cells or senolysis has attracted attention towards combatting such problems. A number of clinical trials are currently underway to investigate innovative pharmacological treatment methods. For example, the therapeutic potential of senescent cell removal has been demonstrated in mouse models of diverse diseases and disorders, including pulmonary fibrosis [2,3], atherosclerosis [4,5], diabetes [6,7], and neurodegeneration [8,9]. In the skin, the senescent phenotype is attributed to senescent cells accumulated in the epidermal and dermal cells and subcutaneous adipose tissue depots, and the development of new senolytic agents is expected [10].

One approach for senescent cell elimination focuses on the fact that senescent cells differ significantly from proliferating cells in the pattern of expressed proteins, including those on the cell surface, which can serve as markers and therapeutic targets. This strategy of using senescent cell-specific markers to target senescent cells is similar to that used to selectively eliminate cancer cells [11]. Senolysis-mimicking anti-cancer therapies targeting specific markers of senescent cells, such as antibody-dependent cellular cytotoxicity [12] and T-cell therapy [13], have been reported. In a previous study, we found that ApoD is specifically expressed in senescent fibroblasts in the dermis [14]. Here, we aimed to develop a senolysis method by combining the targeting of this marker with the existing idea of anti-cancer therapies.

Recently, one of the therapeutic approaches used to target only specific cells is the use of antibody–drug conjugates (ADCs). The therapeutic concept of ADCs is to selectively target tumor cells with small-molecule cytotoxic drugs to maximize cell-killing efficacy and minimize toxicity [15]. ADCs typically consist of antibodies that chemically target proteins on the surface of the target cells. The antibodies bind to the target protein and are generally internalized by the cell. Cytotoxic agents are released into endosomal or lysosomal compartments, and by diffusion or transport, these cytotoxic agents can exert cell-killing effects. Currently, specific ADCs have been developed and are FDA-approved for the treatment of breast cancer, lymphoma, multiple myeloma, and gastric cancer [16,17].

We aimed to analyze whether the specific elimination of senescent fibroblasts occurs when a secondary antibody conjugated with pyrrolobenzodiazepine (PBD), a candidate ADC that inhibits DNA synthesis, is administered using a monoclonal antibody against ApoD, a senescent fibroblast specific marker. This approach could potentially aid in the development of a new method of senolysis.

## 2. Results

### 2.1. ApoD Antibodies Are Specifically Internalized within Aging Dermal Fibroblasts

**Figure 1:**
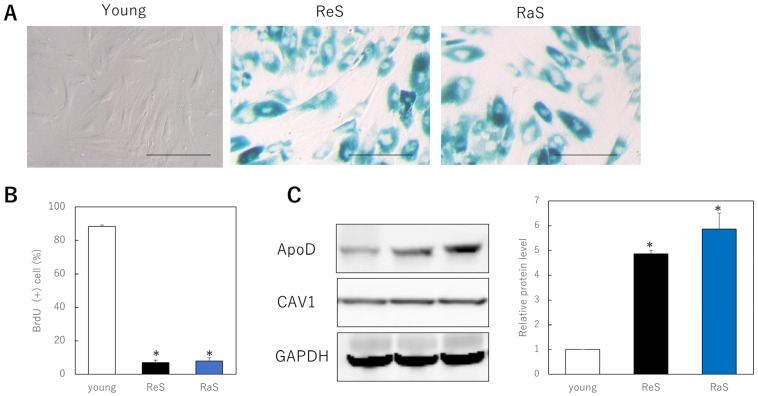

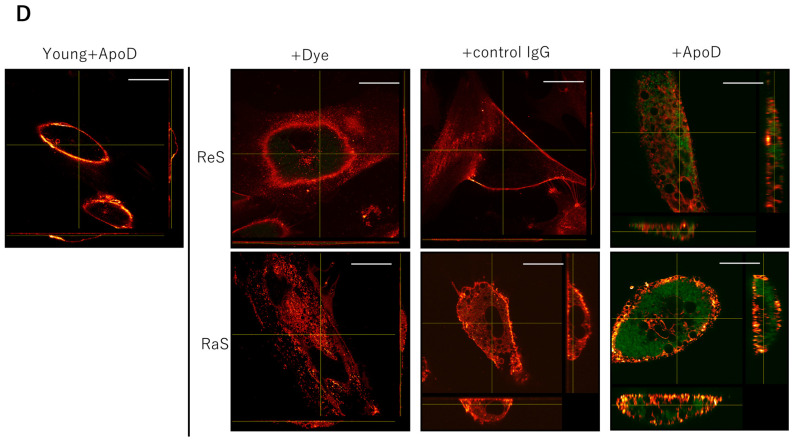
Internalization of ApoD monoclonal antibody in senescent fibroblasts. (**A**): SA-β-gal staining. A large number of positive cells are observed in senescent cells. Bar = 50 μm. (**B**): BrdU uptake in proliferating and senescent cells. *: *p* < 0.05. (**C**): Quantitative comparison of ApoD protein on the plasma membrane in each cell model. *: *p* < 0.05. (**D**): Internalization of fluorescently labeled ApoD antibody in senescent cells. Green fluorescence indicates ApoD internalization, red indicates plasma membrane; Bar = 10 µm; ReS: replicative senescence, RaS: radiation-induced senescence.

To determine whether ApoD monoclonal antibodies are specifically internalized within aging dermal fibroblasts, fluorescently labeled ApoD antibodies were administered to cells, as well as fluorescently labeled non-specific IgG and fluorescent dye as controls. Cells induced to senescence by replicative senescence and ionizing radiation showed an increase in SA-β-gal-positive cells (Figure 1A) and a significant decrease in BrdU uptake, indicating reduced mitotic activity (Figure 1B). In addition, the membrane protein marker CAV1 was found to be expressed at similar levels in all cell models, while ApoD was found to be significantly more highly expressed in the cell membrane of the senescent cell model compared to younger cells (Figure 1C).

When fluorescently labeled antibodies were administered to these cells, fluorescence was observed in the cytoplasm only when the senescent cells were treated with fluorescently labeled ApoD antibodies. This did not occur with the usage of fluorescent dye alone or of a non-specific antibody. Furthermore, no internalization of antibody sites was observed when young cells were treated with ApoD antibodies. The results indicate that the ApoD antibody is specifically taken up and internalized by senescent cells (Figure 1D).

### 2.2. Combination of ApoD Antibody and a Secondary Antibody Conjugated with PBD Specifically Eliminates Human Skin Fibroblasts

**Figure 2:**
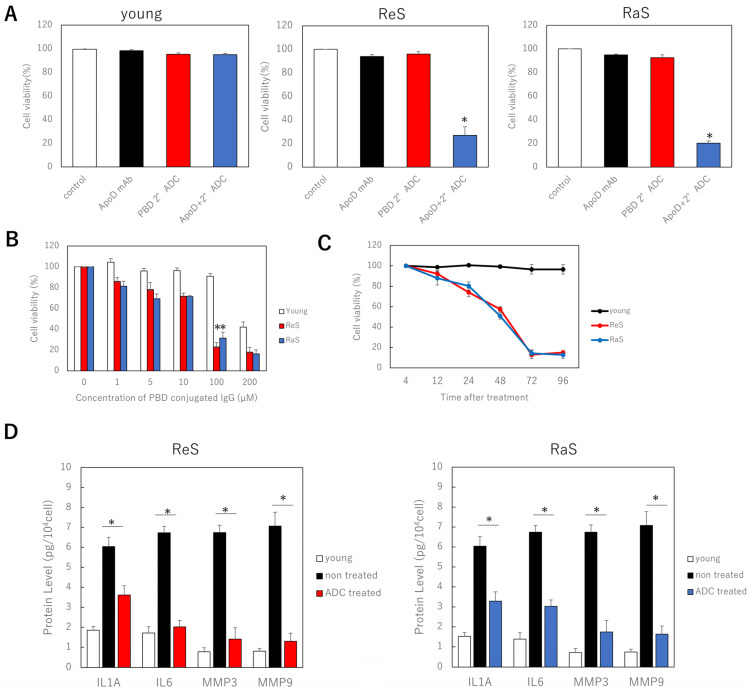
Specific killing of senescent fibroblasts by combination of ApoD antibody and PBD conjugate secondary antibody. (**A**) Cell viability in each cell model with ADC treatment. (**B**): Relationship between concentration of PBD-conjugated secondary antibody and cell viability. (**C**): Relationship between time after ADC treatment and cell viability. (**D**): ELISA assay of the effect of ADC treatment on SASP. ReS: replicative senescence. RaS: radiation-induced senescence. *: *p* < 0.05.

Since the ApoD antibody was found to be specifically taken up and internalized by senescent cells, we investigated whether cell-specific killing occurred when a secondary antibody conjugated with cytotoxic PBD was administered with the ApoD antibody. As controls, we administered PBS (control), a primary antibody only, and a PBD-conjugated secondary antibody only. In young cells, there was no difference in cell viability with either intervention. However, in the two senescent cell models, the combination of primary and secondary antibodies significantly reduced viability (Figure 2A). To optimize the concentration, we administered multiple concentrations of the PBD-conjugated secondary antibody and found a significant difference in survival between young and senescent cells at 100 µM, which is the concentration used in the assay (Figure 2B). Thus, it was shown that senescent fibroblasts are specifically killed by ADC with the ApoD antibody and the PBD conjugated secondary antibody. Furthermore, when the relationship between the time after the start of the antibody treatment and cell viability was investigated, the viability of senescent cells decreased significantly after 72 h, and the extent of the decrease was the same after 96 h; hence, the treatment time was determined to be 72 h (Figure 2C).

In addition, the concentrations of inflammatory cytokines, such as IL6 and IL8, as well as MMP3 and MMP9 proteins involved in dermal senescence, in the medium were reduced after these treatments, indicating that the senescent cell secretory phenotype (SASP) was suppressed (Figure 2D).

### 2.3. Combination of ApoD Antibody and Secondary Antibody Conjugated with PBD Rejuvenates Skin of Aging Mice

**Figure 3:**
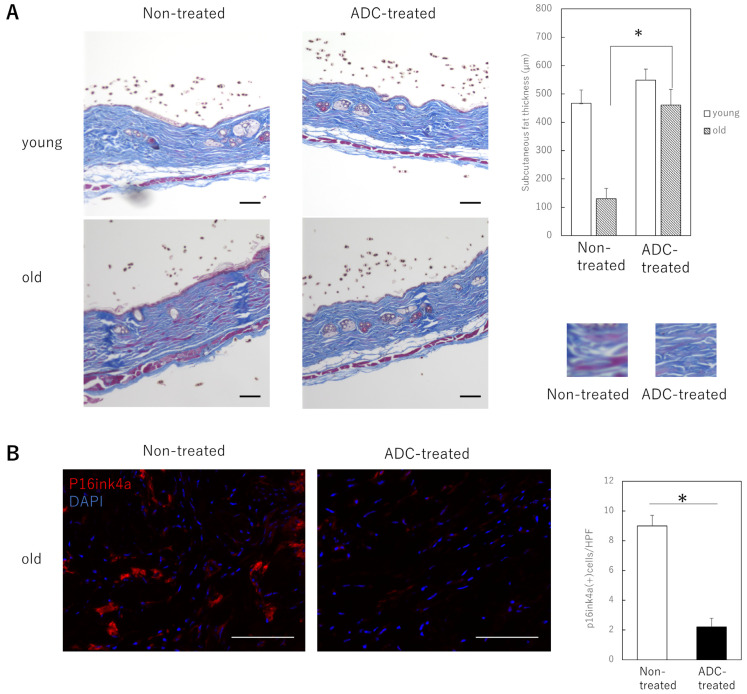
Effects of ApoD-targeted ADC treatment in the skin of young or aging mice. (**A**): Histological changes resulting from treatment of young or aged mice. The image of collagen fibers in the dermis is in the lower right panel. The thickness of the subcutaneous fat in ADC-treated aged mice is significantly increased. Bar = 100 µm. (**B**): Immunofluorescence staining for p16 in tissue sections of mouse skin treated with control vehicle or ADC. Red: p16; blue; DAPI (nucleus). p16-positive dermal cells at a depth of 100 μm from the basement membrane were counted using DAPI. All results are expressed as mean ± SEM. *: *p* < 0.05; bar = 100 μm.

To determine the effect of treatment using a combination of a anti-ApoD primary antibody and a PBD-binding secondary antibody on the skin of animals, young and old mice were administered a single dose of vehicle and the primary and secondary antibody combination intravenously, respectively. The results showed no histological changes in the skin of the young mice, but a significant increase in the thickness of the subcutaneous fat of the old mice was observed. The thickness of collagen fibers in the dermis was also increased by the ADC treatment (Figure 3A). The number of senescent cells (p16ink4a-positive cells) in the dermis was also significantly reduced (Figure 3B). No apparent adverse events, such as death or the appearance of skin ulcers in the mice, were observed during the observation.

## 3. Discussion

Our results show that a monoclonal antibody against ApoD, a marker of aging dermal fibroblasts, was specifically taken up and internalized into the cytoplasm. Furthermore, when a complex of a secondary antibody was conjugated to this monoclonal antibody and a cytotoxic PBD was administered in combination, senescent cell-specific elimination was observed. Senescent cells have been reported to secrete a variety of cytokines (SASP) that affect the microenvironment of the tissue and disrupt its structure and function through a paracrine effect [18,19]. Additionally, treatment with ADCs inhibited the secretion of inflammatory cytokines associated with skin aging, and MMP secretion was inhibited. In an in vivo study, the administration of ADCs in combination with antibodies to aging mice reduced the number of senescent cells in the dermis and thickened collagen fibers without significant adverse events. In addition, the thickness of subcutaneous fat was significantly increased. The decreased collagen fiber thickness and subcutaneous fat thickness was consistent with the aging phenotype, and treatment resulted in improvements in these phenotypes [10]. Overall, our findings support the idea that treatment with a combination of anti-ApoD antibodies and PBD-conjugated secondary antibodies may play a partial role as a novel mechanism of senolysis in aging skin.

Previous studies have indicated that ApoD expression may be induced by stress conditions, such as oxidative and inflammatory stress or UV treatment [20]. The nuclear factor PARP-1 (Poly ADP-ribose polymerase-1), which is upregulated in growth-arrested cells under special circumstances that induce senescence, such as oxidative stress, induces ApoD expression [21,22]. Thus, it is consistent that DNA damage caused by replication or ionizing radiation induces ApoD expression, and serves as an internalizing marker of senescence, as shown in the present study. 

The main cellular source of ApoD-inducing reactive oxygen species (ROS) is the mitochondria, and in a vicious cycle, ROS damage mitochondrial enzymes and mitochondrial DNA, and more ROS are produced due to defects in oxidative phosphorylation reactions [23]. Dysfunction of this intracellular pathway can lead to aging-related diseases, and an approach for ApoD induced by ROS may exert an anti-aging therapeutic effect by interfering with mitochondrial biosynthesis-related pathways [24].

Treatments that selectively destroy senescent cells include ABT263 [25] and ABT737 [26], which inhibit the anti-apoptotic protein Bcl family, as well as dasatinib and quercetin [27] as senolytic drugs that have been reported, but these have been associated with serious side effects. Therefore, attention is needed in finding markers with high specificity for tumor cells in the treatment of melanoma and other cancers to reduce side effects [28]. There is a need for senolysis as a highly specific therapy to kill senescent cells; antibody-dependent cell-mediated cellular cytotoxicity targeting DPP4 [12], antibody–drug conjugates targeting B2M [29], and CAR T therapy targeting the urokinase plasminogen activator receptor (uPAR) [13] were discovered using antigens with high specificity for senescent cells. 

ADCs are based on the recognition by antibodies of extracellular epitopes, which are then internalized, and the drug attached to them is released by the cleavage of linker molecules in lysosomes [30].

Our experiments showed that combination treatment with ApoD monoclonal antibody and PBD-conjugated secondary antibody selectively induced senescent cell death and decreased the expression of senescence-associated SASP without significantly affecting the survival of control proliferating cells. Furthermore, ApoD-negative young cells did not respond to ADC, and isotype control antibodies had no effect on senescent cell survival. This indicates that drug delivery indeed occurs via the specific binding of the antibody to ApoD and does not affect the cells themselves. This information suggests that ADCs can be generated for different targets and can be made specific to cell types or tissues to more selectively eliminate replication or stress-induced senescence, depending on clinical needs.

The ApoD-targeted ADC therapy used herein may be a solid alternative to existing methods as it specifically eliminates senescent cells without affecting younger cells and improves the phenotype of aging skin without apparent side effects.

These proof-of-principle data indicate that ADCs can be effectively used to eliminate senescent cells. However, our study has several limitations, and further experiments are needed to fully understand the relevant mechanisms. Our data strongly suggest that the specific removal of cells can be achieved by the internalization of ADCs. The effects of linker cleavage and the involvement of alternative pathways need to be explored in the future. In addition, no conclusions can be drawn on the aging of other types of cells in the skin, such as keratinocytes and macrophages, the fibroblasts of other tissues, or on side effects. In the present study, no apparent adverse events were observed after in vivo administration to mice. However, the major adverse effects of PBD in patients include vascular leak syndrome, elevated liver enzymes, myelosuppression, gastrointestinal effects (nausea, vomiting, diarrhea, and mucositis/stomatitis), metabolic effects (hyponatremia, hypophosphatemia, etc.), musculoskeletal effects (muscle weakness and myalgia), neuropathy, pain, dyspnea, fatigue, and renal impairment (hyperuricemia and proteinuria) [27]. Therefore, patients will need to be carefully monitored for the appearance of these symptoms with prolonged administration or changes in dosage. However, if necessary, cytotoxic stimuli that require the presence of multiple targets on the cell surface can be designed, which would greatly reduce potential toxic side effects, increase specificity, and increase the feasibility of the approach [31,32].

Our results indicate that specific antibodies may be an efficient system for introducing toxic drugs into human-aged dermal fibroblast cells, following the success of similar approaches in cancer therapy. Further studies are needed to determine the best targets, as well as the safety and efficacy of the therapy, but these data are a potential contribution to the development of new skin rejuvenation therapies.

## 4. Materials and Methods

### 4.1. Cell Culture

Normal human dermal fibroblasts (NHDF) were obtained from Takara Bio (Shiga, Japan). NHDFs were grown in a low-glucose Dulbecco’s modified Eagle medium (Wako Pure Chemical Industries, Osaka, Japan) supplemented with 1% penicillin/streptomycin (Thermo Fisher Scientific, Waltham, MA, USA) and 10% fetal bovine serum (Thermo Fisher Scientific).

Replicative senescence was defined as a cell population doubling level greater than 50 and no proliferation for more than 2 weeks. Ionizing radiation-induced senescence was induced in the same manner as previously reported [14]. Cells were irradiated with 10 Gy of X-rays by AB-160 X-Ray Irradiation System (AcroBio, Tokyo, Japan) and analyzed 10 days later. Control (young; proliferating) cells were mock-irradiated by removal from the incubator, transport to the irradiator, and maintenance outside the irradiator for the same period as the irradiated cells. Intracellular SA-β-gal activity was assessed by staining cells using Senescence β-Galactosidase Staining Kit from Cell Signaling (Danvers, MA, USA).

### 4.2. Evaluation of Proliferative Capacity by Measuring BrdU Uptake

BrdU uptake was assessed using Frontier BrdU Cell Proliferation Assay (Exalpha Biologicals Inc, Shirley, MA, USA) according to the manufacturer’s protocol. SpectraMax i3x (Molecular Devices, San Jose, CA, USA) was used for analysis at 450/550 nm.

### 4.3. Membrane Protein Quantification (Western Blotting) 

To extract membrane proteins from cells, cell lysates were prepared according to the manufacturer’s protocol using the Mem-PER™ plus membrane protein extraction kit (Thermo Fisher Scientific, Waltham, MA, USA) and processed.

Each sample (40 μg) was electrophoresed on 10% polyacrylamide gels (Mini-PROTEAN TGX precast gels; Bio-Rad Laboratories, Inc.) and transferred to polyvinylidene chloride membranes (Millipore, Bedford, MA, USA). After blocking with 3% nonfat milk for 1 h at 25 °C, the membranes were incubated overnight at 4 °C with primary antibodies against apolipoprotein D/APOD (1:100, 347-MSM4-P1; ThermoFisher Scientific), CAV1 (1:1200, sc-53564; Santa Cruz Biotechnology, Santa Cruz, CA, USA) and GAPDH (1:2000; Santa Cruz Biotechnology) in a blocking solution. The next day, the samples were incubated with goat anti-mouse IgG H & L (Horseradish peroxidase) (ab205719; abcam, Cambridge, UK) at 1:1000 dilution for 2 h at 37 °C. After washing, immunoreactive protein bands were visualized using an electrochemiluminescence detection kit (Pierce Biotechnology, Rockford, IL, USA). Images of the bands were acquired using a chemiluminescence imager (ImageQuant LAS4000mini; GE Healthcare, Chicago, IL, USA). Image analysis was performed using ImageJ (ver. 1.53p, National Institutes of Health, Bethesda, MD, USA). Each experiment was repeated three times.

### 4.4. Fluorescence-Labeled ApoD Monoclonal Antibody Uptake Assay

To confirm the endocytosis of mouse anti-human apolipoprotein D/APOD monoclonal antibody (ThermoFisher Scientific) into the cells, Alexa Fluor™ 488 Antibody Labeling Kit (ThermoFisher Scientific) was used to fluorescently label the antibody according to the manufacturer’s protocol. As a control, an anti-human IgG antibody (ab200699, abcam) was prepared with a similarly labeled antibody and Alexa Fluor 488 dye (ThermoFisher Scientific). Aged or normal human skin fibroblasts were plated in 96-well plates (5 × 10^3^ cells per well, *n* = 4) and maintained in 100 μL of a medium. After 24 h, fluorescently labeled the anti-ApoD antibody (final concentration: 10 µM), anti-human IgG (final concentration; 10 µM), and fluorescent dye (final concentration: 10 µM) were added to respective wells. After 24 h, the cells were collected, labeled with CellMask™ Deep Red Plasma Membrane Stain (ThermoFisher Scientific), and observed with the confocal laser scanning microscope FLUO-VIEW FV3000 (Olympus, Co., Ltd. Tokyo, Japan).

### 4.5. Antibody–Drug Conjugate Assay

Aged or normal human skin fibroblasts were plated in 96-well plates (5 × 10^3^ cells/well, *n* = 4) and maintained in 100 μL of a serum-free medium; after 24 h, the anti-ApoD antibody (final concentration: 10 μM) or PBS (control) was added, and the cells were incubated for another 24 h. Anti-mouse IgG (Fc Specific) or PBD-conjugated IgGs with a cleavable linker (Moradec LLC, San Diego, CA, USA) was added at 100 μM, and the cells were incubated for another 72 h. In addition, PBD-conjugated IgG was administered at the concentrations of 0, 1, 5, 10, 50, 100, and 200 μM to optimize the concentration of secondary ADCs. To optimize the reaction time, viability was measured at 4, 12, 24, 48, 72, and 96 h after the administration of PBD-conjugated IgG. Cell viability was analyzed using CellTiter-Glo^®^ 2.0 Cell Viability Assay (Promega, Madison, WI, USA) according to the manufacturer’s protocol. Relative viability was normalized against the PBS control, and quantification experiments were performed in triplicate.

### 4.6. ELISA 

After the preparation of the young and senescent cell model as described above and treatment with ADC and controls, the culture media were collected and subjected to ELISA [Human IL-6 Quantikine ELISA Kit (D6050), Human IL-8 Quantikine ELISA Kit (D 8000C), Human MMP-9 Quantikine ELISA Kit (DMP900) (R & D Systems, Inc., Minneapolis, MS, USA), and Human MMP3 ELISA Kit (ab100607, abcam)] for quantifying IL-6, IL-8, MMP9, and MMP3 concentration.

### 4.7. In Vivo Efficacy Study

Male Bl6 mice (Sankyo Laboratories, Inc., Tokyo, Japan), at 9 weeks old (young) or 80 weeks old (old), were intravenously treated with the vehicle alone or with anti-ApoD antibody and PBD-conjugated IgG with a cleavable linker (Moradec LLC, San Diego, CA, USA), each at a concentration of 0.3 mg/kg and 3 mg/kg in a single dose. Each group contained five mice. The mice were kept with free access to food and water; after 1 month, the mice were euthanized, and tissue samples were collected. The frozen specimens were sliced into 7 µm thick sections, mounted on glass slides, and fixed in acetone for 10 min at room temperature. To block nonspecific binding sites, the slides were incubated with 3% goat serum in PBS for 30 min at room temperature. The slides were then incubated overnight at 4 °C with the primary antibody p16 (ab108349, abcam, 1:200). After washing three times with PBS, the slides were incubated with a Alexa Fluor 488-conjugated goat anti-rabbit antibody (ThermoFisher Scientific) diluted at a ratio of 1:1000 in PBS for 1 h at room temperature. Nuclear contrast staining was performed using ProLongGold with the DAPI anti-fading sealant (ThermoFisher Scientific).

### 4.8. Statistical Analysis

Statistical analyses were performed using GraphPad Prism (version 5.0; San Diego, CA, USA) or SPSS 22.0 (Chicago, IL, USA). A one-way ANOVA and Tukey’s post hoc tests were used to compare the differences between three or more groups. Statistical significance was set at *p* < 0.05.

## Data Availability

The data that support the findings of this study are available from the corresponding author, K.T., upon reasonable request.
